# Influence of root fragility and irrigation on external root temperature variation during preparation for fiberglass posts

**DOI:** 10.4317/jced.60538

**Published:** 2023-11-01

**Authors:** Isabella Göhringer, Maria-Cecília-Lopes Giacomel, Flares-Baratto Filho, Pedro-Henrique Magão, Adilson-Yoshio Furuse, Gisele-Aihara-Haragushiku Furuse, Carla-Castiglia Gonzaga

**Affiliations:** 1 Department of Dentistry, Endodontics and Dental Materials, Faculty of Dentistry of Bauru, University of São Paulo, Bauru, São Paulo, Brazil – Alameda Dr. Octávio Pinheiro Brisolla, 9-75, Bauru, SP, Brasil

## Abstract

**Background:**

This paper analyses the effects of root canal fragility and irrigation on external temperature change (ΔT) of different sections of roots during post-space preparation.

**Material and Methods:**

Forty endodontic treated human premolars were evaluated. Roots were divided into four groups based on their root wall thickness (fragile or non-fragile), and whether they received irrigation (yes or no) during post-space preparation. Initial root canal temperature was kept at 37°C. ∆T was evaluated with thermistors attached to the cervical and apical thirds of the roots during two preparation steps: 1) removal of gutta-percha with Largo drills, and 2) using the specific drill for post-space preparation for cementation of fiber-reinforced posts. In the irrigated groups, we used a 2% chlorhexidine solution during the exchange of drills. ∆T data was analyzed using four-way ANOVA with repeated measures and Tukey’s test (α = 0.05).

**Results:**

Significant differences in ∆T based on root fragility (*p* = 0.017), root canal third (*p* = 0.013), and preparation step (*p* = 0.006). We found that non-fragile roots tended to have higher ∆T than fragile roots, particularly in the apical third, during the use of the second drill. Irrigation did not have a significant effect on temperature variation, regardless of root wall thickness or the third evaluated (*p*> 0.05).

**Conclusions:**

Findings suggest that root wall thickness and the third evaluated influence temperature changes during post-space preparation for cementation of posts. Non-fragile roots showed greater temperature variation than fragile roots, while irrigation did not significantly impact temperature changes.

** Key words:**Temperature, post and core technique, tooth preparation.

## Introduction

When endodontically treated teeth need to receive crowns, posts and cores may be indicated for increasing retention of the prosthetic restorations (1,2) and to better distribute the stress from the masticatory loads toward the root (3-5).

Even though tooth root tissues have poor thermal conductivity (6) heat produced inside the lumen of the canal may partly radiate through thin walls to the outer root surface posing risks to the periodontal ligament when high-speed rotary instruments are used in its interior and there is a temperature variation of +- 10 degrees C (7,8).

However, a clinical aspect that is commonly overlooked during post-space preparation is the temperature variation caused by the use of rotary instruments (9).

The action of these rotary instruments inside the root canal generates an increase in the root temperature. The temperature increase at the external surface of the root has been the subject of studies in the late 80’s but has not been evaluated when specific drills for post-space preparations to receive fiber-reinforced are used. The temperature increase at the external surface of the root approaches 10 ºC above the normal body temperature, which may generate harmful effects such as ankylosis and bone resorption (8). In addition, an *in vivo* study showed that heat generation after post-space preparation generated dental resorption in 27.7% of cases and bone ankylosis in 22.2% of cases (9,10).

Temperature increases can occur due to various factors, such as the force employed by the operator on the root canal walls, the drill type, and diameter, the root anatomy, the amount of remaining dentin thickness, friction time between root canal dentin and drill, the use of refrigeration as well as the use of new or old drills (9,11-14). Studies have shown an increase in external root temperature during post-space preparation (9,11-15), but little is known about the magnitude of temperature increase in teeth with smaller dentin thickness such as fragile roots (13).

Thus, the present study aimed to evaluate the influence of root fragility and irrigation on the variation of root external temperature in the cervical and apical thirds during post-space preparation. The null hypotheses were: I) there would be no influence of root fragility on root external temperature variation during post-space preparation; II) there would be no influence on the external root temperature when irrigating the canal with chlorhexidine during the post space preparation; and III) there would be no differences on external root temperature during post-space preparation regarding root third; IV) there would be no differences on external root temperature during post-space preparation regarding different drills.

## Material and Methods

-Study design

Four factors were evaluated: root fragility (two levels); irrigation between drills exchange (two levels); root canal third (two levels); and preparation step (two levels). The response variable was temperature change (ΔT) in degree Celsius during different preparation steps of the root canal (ΔT during initial gutta-percha removal with Largo drill and ΔT during the use of specific drill for the post-space preparation).

-Sample preparation and ΔT evaluated

Forty monoradicular human premolars with only one root canal extracted for orthodontics reasons were used in this study after approval by the local Institutional Review Board (approval protocol 1.732.273). One calibrated examiner with experience in Endodontics and Prosthodontics performed all procedures. Before the experiments, the pressure necessary for preparation was calibrated with a digital scale and the preparation time for each step was calibrated with a chronometer.

**Figure 1:**
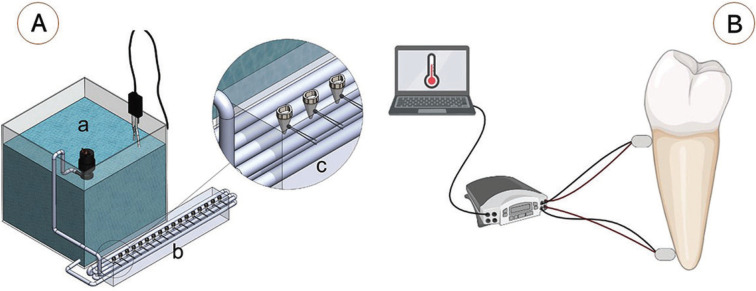
A) Schematics showing an overview of the device assembled to determine the external temperature of the roots. The device is composed of a water reservoir (a) in which the temperature was kept constant at 37ºC. The water of pumped through copper tubes to a lateral circulator (b) which contained spots for fixing each tooth (c). B) After fixing in the circulator, two thermistors were fixed on each tooth (one at the cervical third and the other at the apical third.

Teeth were stored in 0.9% thymol solution at 4ºC right after extraction. Digital radiographs were taken to verify the number of canals, and the exclusion criteria were: the presence of two or more canals and previous endodontic treatment. The teeth that presented complete apexes, root length greater than 14 mm, and absence of previous endodontic treatment had their crowns removed to standardize the root length in 14 mm. The root canals were explored with a #15 file (Dentsply Maillefer, Ballaigues, Switzerland), followed by 13 mm conical preparation of the entire root canal up to 0.60 mm final apical diameter. Irrigation with 2.5 mL of NaOCl 2.5% was performed between file exchanges. Twenty roots were submitted to internal additional enlargement, to simulate fragilized roots, leaving the surrounding root walls with the thickness of 1 mm. This procedure was performed with a 1.2 mm tip diameter conical diamond bur (n. 3169, KG Sorensen, Barueri, SP, Brazil), calibrated 9 mm in length, under copious water irrigation. Digital radiographies were used to measure remaining root wall thicknesses at cervical, medium and apical thirds. The roots were then divided into two groups, according to root wall thickness: non-fragile roots (average root edge wall thickness of 2 mm) and fragile roots (average root edge wall thickness of 1 mm). The sample units were filled with gutta-percha cones (Dentsply Maillefer, Ballaigues, Switzerland) and AH Plus cement (Dentsply Maillefer, Ballaigues, Switzerland), and the condensation of the gutta-percha was performed with lateral condensation technique. Seventy-two hours after the endodontic filling, the roots were subdivided into four groups (n = 10), depending on root wall thickness (fragile or non-fragile) and irrigation mode (yes or no). All roots were fixed in a device developed for the experiment as described below (Fig. 1).

The device is composed of a water reservoir in which the temperature was kept constant at 37ºC controlled by a digital thermometer. The water was pumped through copper tubes to an open rectangular box of 30 x 5 x 10 cm (length x width x high), filled with dental plaster which contained spots for fixing each root. The spots were individually molded for each root to be inserted and being stable. Also, two standardized orifices were made in the box for each specimen at the level of cervical and apical thirds of the root, so that 2 thermistors (10kΩ, NTC, Veteng Thinking, Guangdong, China) were located in intimate contact with the external surface of the roots. The thermistor sensors ran longitudinally and were internalized by the plaster toward the roots while maintaining light pressure, preventing the loss of contact between the tip of the sensor and the external root surface. After fixing the roots in the box, two thermistors were positioned on each tooth, one tooth at a time.

The post-space preparations were carried out with the following times and steps:

• Time between 0 s and 25 s: Partial removal of the gutta-percha with Largo drill, with dry root canal for all groups, performing back and forth movements and touching all the root canal walls, at a speed of 8000 rpm. The speed was kept constant by using an electric micromotor system (Xsmart, Dentsply Maillefer, Ballaigues, Switzerland).

• Time between 25 s and 35 s: Drill exchange and irrigation of the irrigated groups. In the dry groups, the Largo drill was replaced by the #3 drill of a prefabricated glass fiber post kit (DC White Post, FGM, Joinville, SC, Brazil). In the irrigated groups, besides the drill exchange, the roots were internally irrigated with 2% chlorhexidine solution (Maquira, Maringá, PR, Brazil) for 5 s.

• Time between 35 s and 55 s: post-space preparation with the #3 drill for 20 s, performing back and forth movements causing the drill to touch the entire inner surface of the root canal without extra irrigation.

The preparation of the post-spaces was performed with a length of 9 mm, maintaining 4 mm of the apical seal. New drills were used for every 5 specimens. Over the 55 s period, the measurement data generated an exponential curve. With this, it was possible to generate a curve by fourth-degree polynomial interpolation. Data acquisition was obtained through the QuantumX 840A model HBM module (Darmstadt, Germany) and the previously calibrated thermistors utilizing 2-wire connection in DB15 plugs. The equation generated by the interpolation of sensor calibration was programmed in the CatmanEasy@AP software (version 3.1, HBM, Darmstadt, Germany), where each ohmic resistance reading was simultaneously converted to degrees Celsius at a frequency of 50 Hz, providing a measurement every two-hundredths of a second.

During the 55 s of post-space preparation, temperature changes were continuously measured using thermistors located in the cervical and apical thirds of the roots with a sensitivity of 0.1°C. The maximum temperature increases from 0 to 25 s and from 25 to 35 s were recorded. In addition, for each specimen, the pre-preparation baseline temperatures were subtracted from the maximum-recorded temperatures to determine the temperature variation (ΔT) values during each step. Temperature variation data were statistically analyzed with four-way ANOVA with repeated measures and Tukey’s test with a significance level of 0.05. Independent variables were remaining root wall thickness, irrigation, and root canal third. The repeated measure was ΔT of the roots during two preparation steps: 1) during removal of gutta-percha with Largo drills, and 2) when using the specific drill for post-space preparation for cementation of fiber-reinforced posts.

## Results

**Figure 2:**
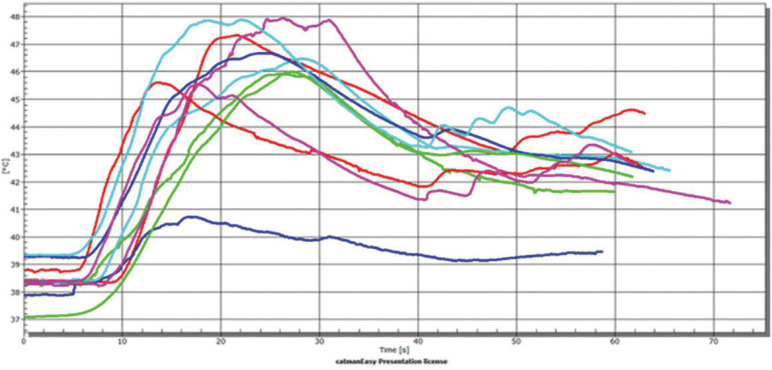
Graph showing the temperature change behavior in the apical third of the non-fragile roots.

In the fragile group, it is possible to notice more clearly the behavior of all steps of the performed procedures. The temperature was sTable (linear) before the procedure, increased during the gutta-percha removal, decreased during the drill exchange period, and increased again when the post-space preparation drill was employed (Fig. 2). The mean values and standard deviations of the temperature variation (∆T) in the two evaluated steps, as a function of fragility, irrigation and thirds are shown in  Table 1. The results indicated statistically significant differences for root fragility (*p* = 0.017), root third (*p* = 0.013) and preparation step (*p* = 0.006). Irrigation was not statistically significant (*p* = 0.655). Regarding double interactions, the following interactions were significant: temperature*00000 (*p* < 0.001); temperature*irrigation (*p* = 0.007). The other double interactions were not statistically significant (*p* > 0.05). Only the triple interaction preparation step*fragility*root third (*p* = 0.003) was statistically significant. The other triple interactions and the quadruple interaction were not statistically significant (*p* > 0.05).

**Table 1:**
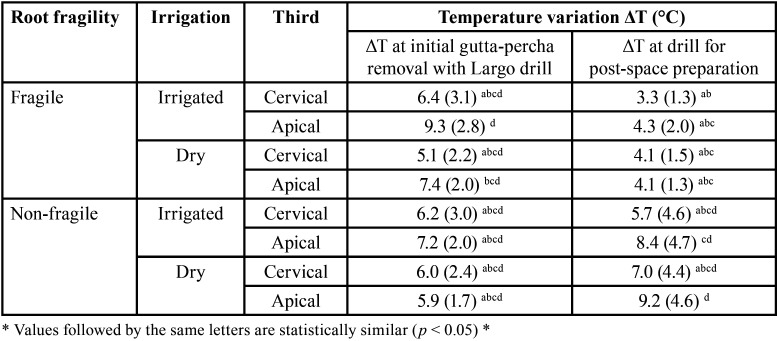
Means and standard deviations of temperature variation (ΔT) in the two preparation steps, as a function of root fragility, irrigation, and root third.

Considering the individual factors, for the root fragility, the non-fragile root groups (6.93 ± 3.7°C) a presented higher ∆T when compared to the fragile root groups (5.5 ± 2.8°C) b. For irrigation, there was no statistically significant difference between the irrigated (6.4 ± 3.5°C) a and dry (6.0 ± 3.1°C) a group. For the root canal third, ∆T was higher for the apical third (6.9 ± 3.3°C) a than cervical third (5.5 ± 3.1°C) b. Regarding the temperature variation in the two different preparation steps, the use of the Largo drill for initial gutta-percha removal (6.7 ± 2.6°C) a presented higher ∆T when compared to the use of the specific drill for post-space preparation (5.7 ± 3.8°C) b.

The preparation step*fragility interaction showed a statistically significant difference for the fragile group at the time of use of the specific drill for post-space preparation, with a lower ∆T (4.0 ± 1.5°C) b than the other groups: non-fragile when using the Largo drill (6.3 ± 2.3°C) a, fragile when using the Largo drill (7.1 ± 2.9°C) a, non-fragile when using the specific drill for post-space preparation (7.5 ± 4.6°C) a. The interaction preparation step*irrigation showed a statistically significant difference, ∆T was lower for the irrigated (5.4 ± 3.9 °C) b and dry (7.3 ± 2.9°C) a group. The groups that were dry presented intermediate and statistically similar ∆T during the use of the Largo drill (6.1 ± 2.2°C) ab and specific drill for post-space preparation (6.0 ± 3.8°C) ab.

In the preparation step*fragility*third interaction, the non-fragile groups did not present larger ∆T than the fragile ones; the apical third presented higher ∆T than the cervical ones; however, there was no difference during the use of the Largo drill and specific drill for post-space preparation.

Observing the data in  Table 1, it can be seen that there was no statistically significant difference in ∆T when using the Largo drill, regardless of the condition of the root, irrigation, or root third. During the use of the specific drill for post-space preparation, the non-fragile roots tended to present higher ∆T than the non-fragile root groups, especially in the apical third.

## Discussion

Endodontically treated teeth suffer extensive loss of crown structure and to be restored may need support. In these cases, additional root canal retention is the alternative of choice through the placement of intracanal posts (1-4,16-18). Even with a changing perspective in dentistry that promotes the replacement of conventional mechanically retained restorations with modern adhesion-dependent methods, these conventional methods of tooth restoration are supported in the literature of long-term follow-up studies to restore endodontically treated teeth with reliability and predictability (16). The dentin thickness is reported in literature as a key factor for performing successful endodontic treatment, since it plays a major role limiting the instrumentation (19) Literature reports that approximately 1 mm of dentin should be present circumferentially in the entire length of the root after root canal instrumentation and post-space preparation to prevent strip perforation and vertical root fracture (19-22). However, it is important that the impacts of temperature elevation resulting from mechanical preparation for cementation of posts be further explored.

The first null hypothesis, that there would be no influence of root fragility on the external root temperature variation during post-space preparation, was rejected since non-fragile roots had significantly higher ∆T when compared to fragile roots. The increase in root surface temperature is caused by several factors such as dentin thickness, friction between the instrument and the wall, and operator-applied pressure when handling the drill (9,11-14,17). Dentin is a good thermal insulator, but heat within the canal can still dissipate into the surrounding tissue as shown in the present study (15,17,25). Literature reports that smaller dentin thicknesses allow more heat to pass to the exterior surface of the root (13). However, in the present study, it is important to emphasize that root fragility (smaller dentin thickness) showed the opposite result. This is due to the fact that, by weakening the root, the dentin thickness decreased, but the post-space increased, reducing the contact of the drill with the walls, thus reducing friction and heat generation. As drills of the same diameter were used in fragile roots or not, the friction may have been reduced due to the increase of the canal’s volume. The fact that the non-fragile roots presented higher temperature variation compared to the fragile ones may be also explained, in part, by the thermoplastic properties of gutta-percha, which would lead to an absorption of part of the energy generated for its thermoplasticization instead of being transferred to the dentin. Consequently, root canals with a larger amount of gutta-percha, such as canals weakened by previous endodontic or prosthetic treatments with increased canal diameter, would have a lower temperature transmission to the external root surface. As mentioned earlier, it can be inferred that the largest temperature variation in the non-fragile root group may be directly related to the greater adaptation and friction with the inner walls during post-space preparation.

The second null hypothesis, that there would be no influence on the external root temperature when irrigating the canal with chlorhexidine during the post-space preparation, was accepted since there was no statistically significant difference between the groups with and without irrigation during the exchange of drills in the post-space preparation. Several studies suggest that post-space preparation should be accompanied by irrigation to minimize root temperature increase. However, the results of this study showed no difference in temperature variation with the use of irrigation. In the previously cited articles, constant irrigation was used throughout the preparation (11,13,14). One of the possible explanations for this result may be the irrigation regime used in this study. Irrigation was performed for a short time (5 s) during the drill exchange to prepare the canal.

The third null hypothesis, that there would be no influence of the root third (cervical or apical on the external root temperature variation during the preparation of the post-space, was rejected. In general, the sensors positioned in the apical third of all groups presented a higher temperature variation compared to the sensors located in the cervical third. As the canal presents a conical shape, with a larger diameter in the cervical third and a smaller diameter in the more apical third, these results can also be explained by the greater friction of the drills in the narrow third, with consequent greater heat generation.

The fourth null hypothesis that there would be no differences on external root temperature during post-space preparation of the roots during the two preparation steps was rejected. Previous studies have shown correlation between root canal instrumentation and temperature variation (22-24), especially when it comes to drills with larger diameters or less pronounced conicities (24). This might be caused by the instrument’s geometry and stiffness that cause excessive forces therefore more friction. As expected, the burr used to prepare the post-space increased the temperature since it has a tapered anatomy especially designed to increase the contact between the burr and the root canal wall.

The temperature variation of the external root surface during the post-space preparation was performed using thermistors. The choice of the thermistor, contrary to methodologies used in previous studies (11,14,25,26) in which thermocouples were used, is justified by the fact that it is a temperature-sensitive device, with the advantage of transmitting the variation of the electric current that will be converted to a temperature quotient in real-time (27). Thermocouples are generally known to be used for high temperatures, whereas thermistors have the best sensitivity and are specific for narrow ranges of temperature, such as the ones used in the medical and dental fields of application (28-30). Also, this is a non-destructive method, since no damage to the specimen is necessary. It should be noted that thermistors must be in close contact with the analyzed area. Therefore, to ensure the best possible contact between the thermistors and the external surface of the roots, the device was designed using individual springs for each of the thermistors, which fit well and individually to each specimen. Another important point to be emphasized is that the device used in the present study was able to keep the specimens close to normal body temperature (37ºC) with the aid of a heat exchanger, making the temperature variation reading from this point, and not from room temperature, as a limitation reported in the literature when infrared readers are used (13,31).

The speed of the drills used in this study was standardized at 8.000 rpm. In addition to speed, the time required to prepare the post-space is also important to minimize tissue damage (9). Although experienced clinicians are able to perform this step quickly, in the present study the short period of 25 s was sufficient to generate a temperature variation above 10°C. There is still no consensus about the threshold temperature for thermal damage to the alveolar bone or periodontal ligament when performing procedures in the root canal. One of the most cited references suggests that an increase in temperature of 10°C on the external root surfaces could lead to bone resorption and dental ankylosis. This classic study reported the occurrence of alveolar bone resorption without signs of regeneration when the bone temperature increased by 10°C for 5 min or 13°C for 1 min (12). However, even lower temperature thresholds have been reported. One study suggested that an increase in temperature of 6°C (~43°C) at the periodontal ligament would be sufficient for the occurrence of protein denaturation, ankylosis, and alveolar bone resorption. In addition, it is important to note that damage to periodontal tissue can occur when the amount of dentin remaining is less than 1 mm (32).

Some points regarding the method should be discussed. Post-space preparation was performed as an attempt to simulate a clinical situation by keeping a water bath at the temperature of 37°C (33,34). The samples were fixed in plaster support, adapted around the roots, simulating the bone insertion. The post-space preparation was performed using a clinically simulated pressure, avoiding the use of excessive force that could interfere in the temperature variation measurements. New drills were used and were changed every five preparations, also in an attempt to avoid excessive temperature increases. In the present study, the remaining dentin thickness was evaluated by digital radiographies. While this is a simple low-cost straightforward method other method such as cone-beam computed tomography scanning could be also be used (35). The use of CT scans could in fact provide more detailed information regarding the 3D morphology of the root. However, for the purpose of our study digital x-rays are proper for evaluation as it has been shown the measuring tools provided by the manufacturers are appropriate and worldwide used for addressing the dimension of root canals during either the root canal treatment or the post space preparation (36).

A previous study measured the *in vivo* temperature during different stages of post-space preparation walls in five patients. The measurements were made in the following times: after the removal of gutta-percha, with the canal filled with saline solution and rinsed, after the placement of paper points, and after 1.5 minutes (period to prepare the luting materials). Significant differences were observed when the treatment stages were compared. As expected, post-space preparation presented the highest temperature, while the lowest was obtained after rinsing (22). It is important to remember that the device used here is not equivalent to actual clinical situations, considering the periodontal tissues, bone circulation, and the oral environment. In some specimens, temperature variations above 10 °C were observed, although no average reached this critical value. Therefore, clinicians should always consider, when preparing root canals for posts, the use of refrigeration, intermittent movements, new drills, and application of controlled force during post-space preparation to avoid excessive temperature and damage to adjacent tissues.

## Conclusions

The root fragility and the root third influenced the temperature variation during post-space preparation for fiberglass posts. Irrigation did not affect on temperature variation. Root fragility positively influenced, with less temperature variation, when compared to the non-fragile root groups. The cervical third presented less temperature variation than the apical third. Finally, the use of the Largo drill presented a higher temperature range of values when compared to the use of a specific drill for post-space preparation.
